# Genome-wide changes accompanying knockdown of fatty acid synthase in breast cancer

**DOI:** 10.1186/1471-2164-8-168

**Published:** 2007-06-12

**Authors:** Lynn M Knowles, Jeffrey W Smith

**Affiliations:** 1 Cancer Research Center, Burnham Institute for Medical Research, La Jolla, CA 92037, USA

## Abstract

**Background:**

The lipogenic enzyme fatty acid synthase (FAS) is up-regulated in a wide variety of cancers, and is considered a potential metabolic oncogene by virtue of its ability to enhance tumor cell survival. Inhibition of tumor FAS causes both cell cycle arrest and apoptosis, indicating FAS is a promising target for cancer treatment.

**Results:**

Here, we used gene expression profiling to conduct a global study of the cellular processes affected by siRNA mediated knockdown of FAS in MDA-MB-435 mammary carcinoma cells. The study identified 169 up-regulated genes (≥ 1.5 fold) and 110 down-regulated genes (≤ 0.67 fold) in response to knockdown of FAS. These genes regulate several aspects of tumor function, including metabolism, cell survival/proliferation, DNA replication/transcription, and protein degradation. Quantitative pathway analysis using Gene Set Enrichment Analysis software further revealed that the most pronounced effect of FAS knockdown was down-regulation in pathways that regulate lipid metabolism, glycolysis, the TCA cycle and oxidative phosphorylation. These changes were coupled with up-regulation in genes involved in cell cycle arrest and death receptor mediated apoptotic pathways.

**Conclusion:**

Together these findings reveal a wide network of pathways that are influenced in response to FAS knockdown and provide new insight into the role of this enzyme in tumor cell survival and proliferation.

## Background

Up-regulation of fatty acid synthase (FAS), the enzyme responsible for the endogenous synthesis of palmitate, is increasingly recognized as a hallmark of cancer [1,2]. While normal cells obtain most fatty acids from circulating lipids, tumor cells have developed an increased reliance on endogenous fatty acid synthesis to satisfy their metabolic needs [2]. This elevation of FAS occurs early in human cancer, is associated with aggressive forms of the disease, and is linked to poor prognosis [3-7]. Consequently, strategies to target FAS are becoming increasingly exploited as attractive approaches for cancer therapy.

FAS is critically important for tumor cell survival and function; providing the necessary fatty acids for membrane formation and signal transduction [2]. The targeted knockdown of tumor FAS by small molecule inhibitors or small interfering RNA (siRNA) has been shown to induce both cell cycle arrest and apoptosis in cultured cells and suppresses tumor growth in xenograft bearing mice [8-13]. This anti-tumor activity is linked to increased expression of p27^kip1 ^[9,14] and decreased Akt phosphorylation [15]. FAS inhibition can also lead to the transcriptional suppression of the *Her2/neu *gene suggesting an active role for FAS in gene regulation [16]. However, detailed understanding of the regulatory mechanisms linking inhibition of FAS to these anti-tumor effects remains elusive.

In the present study, we utilized siRNA, BeadArray technology, and pathway analysis to define the genome-wide changes that take place following knockdown of FAS (12–48 h). We have identified a core set of 279 genes representing the FAS knockdown signature in the MDA-MB-435 mammary carcinoma cell model. Functional classification of these target genes, combined with quantitative pathway analysis, revealed extensive changes in metabolism, cell survival/proliferation, DNA replication/transcription, and ubiquitin dependent protein degradation as a consequence of FAS inhibition. Taken together, our results provide a detailed overview of the anti-tumorigenic signaling network induced in tumor cells by the targeted knockdown of FAS.

## Results

### Changes in gene expression resulting from knockdown of FAS

**Figure 1:**
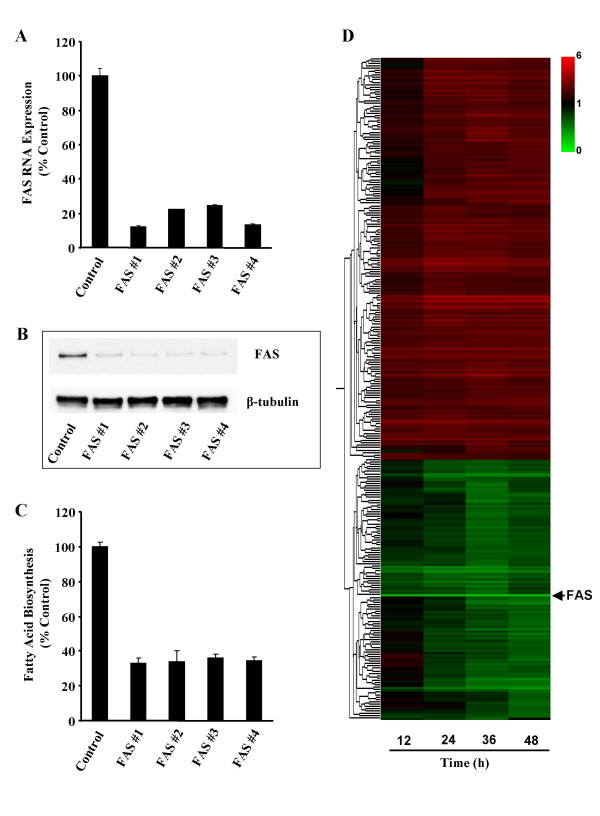
**FAS knockdown generates specific and time-dependent gene expression patterns**. (**a-c**) Target mRNA, protein and fatty acid biosynthesis knockdown by FAS siRNA duplexes. MDA-MB-435 tumor cells were exposed to 25 nM of four different siRNA duplexes targeted against FAS (FAS #1-#4) or non-silencing control siRNA for 48 h. Efficiency of FAS knockdown was determined by measuring FAS mRNA (**a**), FAS protein (**b**) and fatty acid biosynthesis (**c**). β-tubulin served as a loading control for FAS protein expression. Values are the mean ± SE of two replicates per treatment. (**d**) Expression profile represents 279 genes differentially modified by 1.5 fold in response to knockdown of FAS (shown in hours on the X-axis). Horizontal lines represent the average expression of individual genes modified by at least 3 of the FAS siRNA duplexes. Red and green indicate increased and decreased expression, respectively, relative to non-silencing control siRNA.

MDA-MB-435 mammary carcinoma cells were selected as the model for defining the FAS knockdown signature. For our experiment, four independent siRNA duplexes targeting FAS (FAS #1-#4) were chosen based on there ability to knockdown the enzyme and to induce tumor cell apoptosis after 72 h [17]. Inhibition of FAS by each duplex was verified using 25 nM of siRNA as demonstrated by a decrease in FAS mRNA, protein, and fatty acid biosynthesis after 48 h relative to non-silencing control siRNA (Figure 1a–c). The non-silencing control siRNA was selected based on minimal cross reactivity with known targets and had no impact on FAS expression or activity when compared against Lipofectamine 2000 transfection alone (data not shown). Gene expression profiles were examined on two separate occasions following transfection with FAS siRNA. Treatment times (12, 24, 36 and 48 h) were chosen to capture early and late gene changes associated with the block in cell cycle progression and the advent of apoptosis occurring in response to knockdown of FAS. Abrogation of FAS (>70%) was verified within 12 h of transfection and persisted throughout the 48 h experiment. Significant changes in cell viability were not observed 48 h post-transfection indicating cells were viable during the time of gene analysis (data not shown).

The first approach we used in analyzing the effect of knockdown of FAS on gene expression was to identify target genes modified by at least 3 of the siRNA duplexes. The expression signature of each FAS siRNA was determined by identifying genes significantly changed over the time course using *MB *statistics and two way ANOVA analysis. For each siRNA duplex only genes with a *p*-value ≤ 0.05 and at least 1.2 fold changes in both biological replicates at a given time point were maintained for expression analysis, and are published as supporting information online (see Additional Files 1, 2, 3, and 4). The FAS knockdown signature was defined as the overlap of significant gene changes occurring in response to at least 3 of the siRNA duplexes with an average gene expression change 1.5 fold that observed in controls. This allowed us to identify gene changes specifically associated with knockdown of FAS, while at the same time eliminate potential off-target effects unique to the individual siRNA sequences. Using this approach, we identified 279 genes whose expression changed in response to knockdown of FAS (169 genes up-regulated and 110 down-regulated; see Additional File 5). Alterations in gene expression occurred as early as 12 h after knockdown of FAS with the majority of genes affected by 24 h (Figure 1d). These results show that inactivation of FAS has a profound affect on gene transcription.

Knockdown of FAS affected genes that regulate a variety of biological processes including cell proliferation, DNA replication, transcription and apoptosis (Table 1; see Additional File 5). We found that the abrogation of FAS enhanced the expression of several anti-proliferative genes including *CCNG1, CDKN1A *(*p21*^*cip*1^), *SPRY2 *and *SPRY4*. This coincided with up-regulation of the cell surface apoptosis gene *ANXA1*, the mitochondrial apoptosis pathway gene *APAF1*, and the death receptors *TNFRSF10B *(TNF-related apoptosis-inducing ligand (TRAIL) receptor) and *TNFRSF21 *(TNF-α receptor). In addition, results showed knockdown of FAS up-regulates *SGPL1*, a key enzyme in sphingosine-1-phosphate metabolism whose increased expression also leads to accumulation of the pro-apoptotic signaling molecule ceramide [18]. This finding is consistent with reports that FAS inhibition up-regulates ceramide synthesis, which has been found to be a necessary step leading to the induction of tumor cell apoptosis following the loss of FAS [17].

**Table 1:** Selected FAS target genes involved in proliferation/apoptosis, metabolism, transcription and protein ubiquitination.

**Gene Classication/Description**	**Symbol**	**12 h Fold Δ**	**24 h Fold Δ**	**36 h Fold Δ**	**48 h Fold Δ**	**Gene Identifier**
**Proliferation**						
**Up-regulated**						
Cell division cycle associated 4	CDCA4	1.43	1.63	1.82	1.59	GI_22027510-A
Cyclin G1	CCNG1	0.94	1.55	1.41	1.37	GI_40805830-S
Cyclin-dependent kinase 2	CDK2	1.14	1.60	1.43	1.39	GI_16936529-A
Cyclin-dependent kinase inhibitor 1A (p21, Cip1)	CDKN1A	1.60	1.60	1.84	1.82	GI_17978496-A
Polo-like kinase 1	PLK1	1.24	1.35	1.48	1.59	GI_34147632-S
Sprouty homolog 2	SPRY2	1.39	1.68	1.60	1.58	GI_22209007-S
Sprouty homolog 4	SPRY4	1.08	1.43	1.45	1.56	GI_23308573-S
Sprouty-related, EVH1 domain containing 1	SPRED1	1.18	1.36	1.59	1.47	GI_22749220-S
WEE1 homolog	WEE1	1.37	1.55	1.65	1.67	GI_19718775-S
**Apoptosis**						
**Up-regulated**						
Annexin A1	ANXA1	1.29	1.93	1.90	1.63	GI_4502100-S
Apoptotic protease activating factor	APAF1	1.28	1.72	1.48	1.39	GI_32483362-A
CSE1 chromosome segregation 1-like	CSE1L	1.05	1.46	2.40	2.07	GI_29029560-I
P21 (CDKN1A)-activated kinase 2	PAK2	1.13	1.44	1.46	1.71	GI_32483398-S
Sphingosine-1-phosphate lyase 1	SGPL1	1.42	1.55	1.67	1.72	GI_31982935-S
Tumor necrosis factor receptor superfamily, member 10b	TNFRSF10B	1.32	1.81	1.76	1.49	GI_22547118-A
Tumor necrosis factor receptor superfamily, member 21	TNFRSF21	1.20	1.71	1.88	1.73	GI_23238206-S
**Metabolism**						
**Up-regulated**						
Insulin induced gene 1	INSIG1	1.51	1.51	1.56	1.51	GI_38327530-A
Leptin receptor	LEPR	1.30	1.60	1.63	1.59	GI_41327153-S
Leptin receptor overlapping transcript-like 1	LEPROTL1	1.11	1.62	1.59	1.45	GI_7662509-S
**Transcription**						
**Up-regulated**						
E2F transcription factor 7	E2F7	1.26	1.32	1.50	1.47	GI_44955909-S
Early growth response 1	EGR1	1.56	1.56	1.84	1.82	GI_31317226-S
Far upstream element (FUSE) binding protein 1	FUBP1	1.15	1.12	1.51	1.52	GI_17402899-S
FBJ murine osteosarcoma viral oncogene homolog B	FOSB	1.44	1.40	1.66	1.78	GI_5803016-S
FOS-like antigen 1	FOSL1	1.11	1.44	1.65	1.59	GI_34734076-S
Kruppel-like factor 4 (gut)	KLF4	1.04	1.47	1.61	1.36	GI_34916057-S
Meis1, myeloid ecotropic viral integration site 1 homolog	MEIS1	1.12	1.50	1.33	1.30	GI_45006902-S
Nuclear factor I/A	NFIA	1.13	1.40	1.41	1.52	GI_30840979-S
Nuclear factor I/B	NFIB	2.10	2.24	2.29	2.16	GI_5031940-S
Nuclear factor of activated T-cells, cytoplasmic, calcineurin-dependent 3	NFATC3	1.31	1.52	1.48	1.50	GI_27886558-A
Paired related homeobox 1	PRRX1	0.96	1.35	1.59	1.59	GI_12707576-A
Protein phosphatase 1, regulatory (inhibitor)	ELF2	1.30	1.34	1.54	1.41	GI_42544175-A
Serum response factor	SRF	1.55	1.44	1.35	1.26	GI_4507204-S
SP110 nuclear body protein	SP110	1.02	1.25	1.33	1.63	GI_17986251-I
Zinc finger protein 106 homolog	ZFP106	1.33	1.58	1.66	1.68	GI_11968022-S
Zinc finger protein 267	ZNF267	1.25	1.53	1.75	1.52	GI_24431954-S
Zinc finger protein 35 (clone HF.10)	ZNF35	1.55	1.70	1.60	1.58	GI_21361560-S
Zinc finger protein 426	ZNF426	1.36	1.52	1.37	1.35	GI_13129115-S
Zinc finger protein 503	ZNF503	1.28	1.53	1.37	1.28	GI_34222201-S
Zinc finger protein 539	ZNF539	1.04	1.50	1.29	1.13	GI_4758513-S
Zinc finger protein 559	ZNF559	1.48	1.76	1.59	1.53	GI_23618925-S
Zinc finger protein 586	ZNF586	1.73	1.63	1.63	1.51	GI_8923076-S
Zinc finger protein 91 homolog	ZFP91	1.13	1.42	1.60	1.26	GI_25777699-I
**Down-regulated**						
Aryl-hydrocarbon receptor nuclear translocator 2	ARNT2	0.85	0.79	0.62	0.76	GI_41281514-S
CAMP responsive element binding protein 3-like 4	CREB3L4	0.92	0.66	0.66	0.76	GI_31542090-S
Hypothetical protein FLJ37970	FLJ37970	1.08	0.79	0.81	0.67	GI_40795670-S
Inhibitor of DNA binding 2, dominant negative helix-loop-helix protein	ID2	1.00	0.79	0.61	0.71	GI_33946335-S
Inhibitor of DNA binding 3, dominant negative helix-loop-helix protein	ID3	0.93	0.69	0.57	0.56	GI_32171181-S
Neuronal PAS domain protein 1	NPAS1	0.90	0.82	0.71	0.64	GI_22027481-S
Oligodendrocyte transcription factor 1	OLIG1	0.78	0.83	0.69	0.68	GI_41281694-S
Signal transducer and activator of transcription 2, 113 kDa	STAT2	0.90	0.86	0.64	0.71	GI_38202247-S
**Ubiquitination**						
**Up-regulated**						
Ubiquitin-conjugating enzyme E2 variant 1	UBE2V1	1.79	2.54	2.45	2.11	GI_40806192-S
Ubiquitin-conjugating enzyme E2 variant 1	UBE2V1	1.65	1.73	1.83	1.67	GI_40806191-I
Ubiquitin-conjugating enzyme E2E 3 (UBC4/5 homolog)	UBE2E3	1.62	2.21	2.27	2.14	GI_33359693-A
Ubiquitin-conjugating enzyme E2C	UBE2C	1.09	1.11	1.52	1.25	GI_32967282-I
CDC23 (cell division cycle 23, yeast, homolog)	CDC23	1.24	1.75	1.67	1.60	GI_16554575-S
Ring finger protein 144	RNF144	1.52	1.52	1.47	1.51	GI_38045937-S
Makorin, ring finger protein, 1	MKRN1	1.41	1.75	1.64	1.49	GI_21359891-S
F-box and leucine-rich repeat protein 12	FBXL12	1.56	1.81	1.55	1.61	GI_8923178-S
F-box and leucine-rich repeat protein 7	FBXL7	1.52	1.62	1.64	1.68	GI_21071079-S
Ubiquitin-like, containing PHD and RING finger domains, 1	UHRF1	1.15	1.55	1.36	1.35	GI_16507203-S
APG12 autophagy 12-like	APG12L	1.32	1.27	1.54	1.24	GI_38261968-S
**Down-regulated**						
Membrane-associated ring finger (C3HC4) 2	MARCH-II	0.99	0.72	0.60	0.59	GI_31543081-S
SH3 domain containing ring finger 2	SH3RF2	1.00	0.89	0.84	0.66	GI_22749146-S
Tripartite motif-containing 2	TRIM2	0.78	0.83	0.60	0.76	GI_15011942-S
Tripartite motif-containing 37	TRIM37	0.75	0.64	0.64	0.70	GI_15147332-S

The fold change values represent the average from at least 3 of the FAS siRNA duplexes compared to non-silencing control siRNA. Genes were classified using GO terminology from GeneSpring and the NCBI Nucleotide database. For detailed annotations of all 279 differentially expressed genes see Additional File 5.

Several of the genes significantly altered by knockdown of FAS are known to play a role in the regulation of lipid metabolism. Inactivation of FAS increased the expression of *INSIG1*, and the leptin receptors, *LEPR *and *LEPROTL1*, which block transcription, proteolytic cleavage and transcriptional activation of the sterol regulatory element-binding protein (SREBP) family of transcription factors that promote lipid biosynthesis (Table 1) [19,20]. Additionally, loss of FAS led to down-regulation of *ID2 *and *ID3*, which are dominant negative helix-loop-helix (HLH) proteins that bind SREBP 1c and functionally repress FAS promoter activity [21]. ID2 and ID3 also repress expression of the cyclin-dependent kinase inhibitor p21^cip1 ^[22,23], thus, as expected the suppression of these genes in response to knockdown of FAS was coupled with up-regulation of the *p21*^*cip*1 ^gene (Table 1). These data provide evidence of a direct link between regulation of FAS and control of cell cycle progression.

Another important biological category affected by knockdown of FAS is regulation of protein ubiquitination (Table 1). We found that knockdown of FAS altered the expression of several E2 ubiquitin conjugation enzymes (2 up-regulated) and E3 ubiquitin ligases (4 down-regulated and 6 up-regulated) which function to target proteins for degradation by the proteosome [24]. In addition, we identified up-regulation of 2 splice variants of *UBE2V1*, which are catalytically inactive E2 enzymes [25]. UBE2V1 variants result from the co-transcription of *UBE2V1 *with the neighboring upstream gene, *Kua*, which shares sequence consensus to fatty acid hydrolases (variant 2, *Kua-UEV *fusion gene [GI_40806191-I]; variant 4, *Kua *gene [GI_40806192-I]). This targeting of ubiquitination enzymes indicates that the knockdown of FAS affects the tumor cell proteome not only through changes in transcription, but also on the post-translational level.

### Knockdown of FAS leads to widespread changes in pathways involved in metabolism

**Figure 2:**
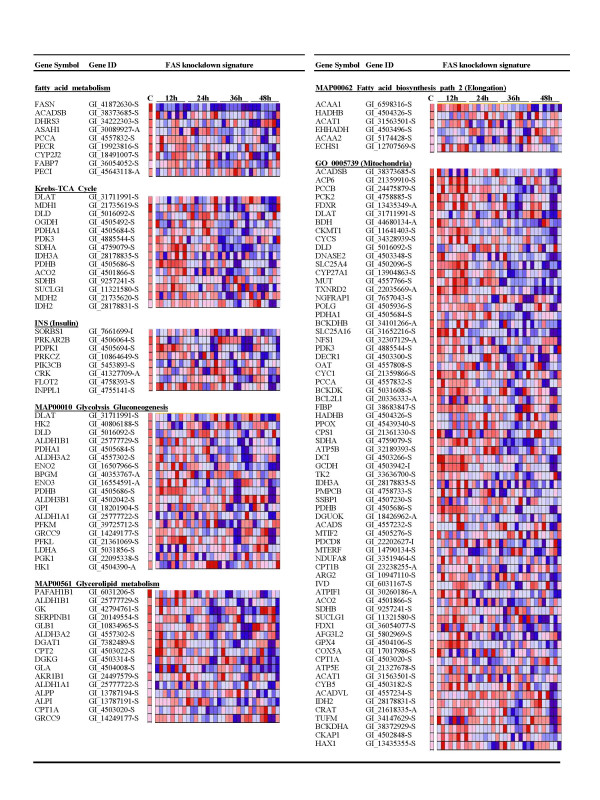
**Down-regulation of metabolic pathways by targeted knockdown of FAS**. The figure displays select pathways found to be down-regulated in response to FAS siRNA treatment compared to non-silencing control siRNA. Significance was determined using a nominal *p*-value < 0.05 or FDR < 0.250. The expression levels of the genes significantly modified in the pathway are coded colorimetrically: red, high expression; blue, low expression. FAS siRNA treatments for each time point are ordered as follows (a and b indicate different biological replicates): FAS #1a, FAS #1b, FAS #2a, FAS #2b, FAS #3a, FAS #3b, FAS #4a, FAS #4b. For a complete list of all pathways down-regulated by knockdown of FAS, see Additional File 6.

The second approach we used to analyze the gene array results was to identify gene pathways coordinately up-regulated or down-regulated by knockdown of FAS using Gene Set Enrichment Analysis (GSEA). GSEA is a unique application that coordinates small changes in gene expression from a large number of functionally related genes in order to identify pathways that are significantly changed [26]. As expected, fatty acid metabolism (NES = -1.808, NOM *p*-val = 0.002, FDR q-val = 0.085) was significantly suppressed by knockdown of FAS (Figure 2). Fatty acid elongation (NES = -1.537, NOM *p*-val = 0.037, FDR q-val = 0.394) was also reduced, which is consistent with the loss of available palmitate.

The effects of knockdown of FAS on the expression of metabolic genes extended to pathways other than fatty acid metabolism (Figure 2). Inactivation of FAS down-regulated both the glycolysis/gluconeogenesis (NES = -1.560, NOM *p*-val = 0.029, FDR q-val = 0.377) and krebs-TCA cycle (NES = -1.737, NOM *p*-val = 0.014, FDR q-val = 0.094) pathways. Down-regulation of the krebs-TCA cycle was consistent with an overall suppression in mitochondrial genes involved in energy metabolism and oxidative phosphorylation (GO 005739; NES = -1.515, NOM *p*-val = 0.039, FDR q-val = 0.365). Furthermore, this down-regulation in genes regulating glucose utilization was coupled with a reduction in insulin signaling (NES = -1.635, NOM *p*-val = 0.005, FDR q-val = 0.280). This suggests that inhibition of FAS may restrict the ability of tumor cells to produce the energy necessary to thrive.

### Knockdown of FAS up-regulates pathways involved in cell cycle arrest and apoptosis

**Figure 3:**
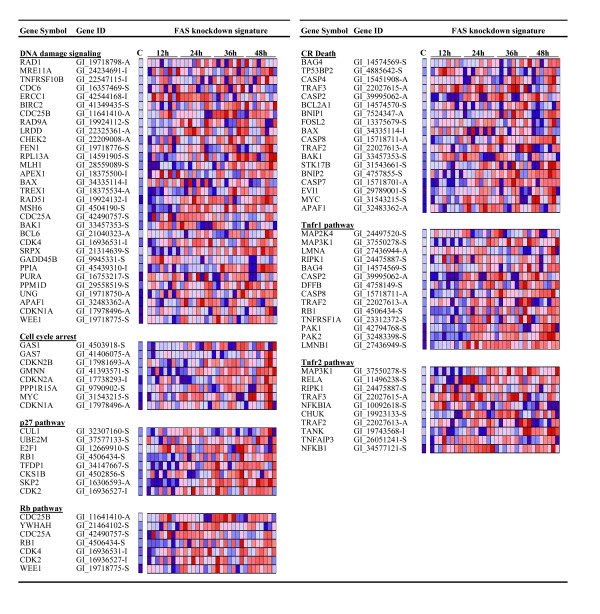
**Up-regulation of cell cycle arrest and cell death pathways in response to knockdown of FAS**. The figure displays select pathways found to be up-regulated in response to FAS siRNA treatment compared to non-silencing control siRNA. Significance was determined using a nominal *p*-value < 0.05 or FDR < 0.250. The expression levels of the genes significantly modified in the pathway are coded colorimetrically: red, high expression; blue, low expression. FAS siRNA treatments for each time point are ordered as follows (a and b indicate different biological replicates): FAS #1a, FAS #1b, FAS #2a, FAS #2b, FAS #3a, FAS #3b, FAS #4a, FAS #4b. For a complete list of all pathways up-regulated by knockdown of FAS, see Additional File 7.

The anti-tumorigenic effects of knockdown of FAS were traced to an up-regulation in genes that modify tumor proliferation (Figure 3). GSEA analysis showed that knockdown of FAS up-regulated genes involved in DNA damage signaling (NES = 1.809, NOM *p*-val = 0.000, FDR q-val = 0.068) and cell cycle arrest (NES = 1.504, NOM *p*-val = 0.036, FDR q-val = 0.163). Induction of cell cycle arrest was coupled with up-regulation of the p27 (NES = 1.582, NOM *p*-val = 0.009, FDR q-val = 0.128) and Rb (NES = 1.581, NOM *p*-val = 0.010, FDR q-val = 0.126) pathways consistent with our previous finding that FAS regulates the G_1 _checkpoint through these pathways [9]. Additionally, knockdown of FAS elevated the expression of several cyclin-dependent kinase inhibitors (*p16INK4 *(CDKN2A), *p15INK4 *(CDKN2B) and *p21*^*Cip*1^) which function as negative regulators of the Rb pathway [27], demonstrating FAS induces a high degree of regulation over the G_1_/S transition (Figure 3). Results also showed that knockdown of FAS elevated genes involved in the G_2 _pathway including the checkpoint regulators *CHEK2*, *WEE1 *and *PLK1 *(Table 1; Figure 3). These data indicate that FAS controls tumor proliferation by regulating several aspects of the cell division cycle.

The apoptotic effects of knockdown of FAS were recently shown to be blocked by co-treatment with the pan-caspase inhibitor z-VAD-fmk [28], suggesting a role for caspases in mediating FAS induced tumor cell death. Here, we found up-regulation of caspase 7 and caspase 8 (receptor-mediated apoptosis) in response to knockdown of FAS (Figure 3). This was accompanied by enhanced expression of pathways and genes that activate caspase 8 such as tumor necrosis factor (tnfr1, NES = 1.408, NOM *p*-val = 0.038, FDR q-val = 0.230; tnfr2, NES = 1.788, NOM *p*-val = 0.002, FDR q-val = 0.070; Figure 3). GSEA results also demonstrated compensatory up-regulation of NF-κB (NF-κB Induced, NES = 1.5, NOM *p*-val = 0.022, FDR q-val = 0.166) and the MAP kinase (NES = 1.539, NOM *p*-val = 0.016, FDR q-val = 0.133) survival pathways in line with previous reports (see Additional File 7; [14]).

## Discussion

The present study provides a comprehensive overview of the genomic changes accompanying knockdown of tumor FAS. To our knowledge, this is the first report demonstrating that targeted knockdown of a metabolic enzyme can influence overall energy producing pathways within the tumor cell. The major findings of this work are as follows: first, siRNA mediated knockdown of FAS affects the transcription of genes involved in tumor cell energy metabolism as demonstrated by down-regulation in lipid metabolism, glycolysis, krebs-TCA cycle and oxidative phosphorylation pathways. Second, we show that loss of FAS is anti-tumorigenic by up-regulating cell cycle arrest and death receptor mediated apoptosis pathways. Third, the inhibition of FAS leads to changes in genes that regulate transcription and ubiquitin-dependent protein degradation. Altogether, these findings provide novel insight into the cellular processes affected by knockdown of FAS in tumor cells.

A vital assumption in siRNA-mediated gene silencing is that knockdown of the target gene is exclusive, and without off-target consequences. Yet, increasing evidence has suggested siRNA can induce off-target effects that are unique to the individual siRNA duplex, but unrelated to the identity of the target gene [29]. These siRNA duplex specific off-target effects are thought to arise from unintended sequence homology with the off-target transcript. In this study, we addressed the concern of potential siRNA off-target effects by examining gene expression signatures in response to four siRNA duplexes targeting different regions of the FAS gene and focused our analysis on the common gene changes that were associated with knockdown of the enzyme. This eliminated off-target effects and enabled us to study gene changes specifically associated with knockdown of FAS. Altogether, evidence supporting the role of FAS inhibition in inducing the described genomic effects is as follows: first, we showed that FAS siRNA strongly abrogates the expression of the FAS gene, reduces FAS protein levels and blocks the biosynthetic activity of the enzyme, as indicated by reduced [^14^C]malonyl-CoA incorporation into palmitate. Second, pathway analysis demonstrated that fatty acid biosynthesis and metabolism are among the most significant pathways down-regulated in response to FAS siRNA. Third, up-regulation of the p27 and Rb gene pathways is consistent with our prior work showing knockdown of FAS elicits a G_1_/S arrest through these pathways [9]. We also found increased expression of the *p21*^*Cip*1 ^gene which is up-regulated at the protein level in response to knockdown of FAS [14,30]. Like p27, p21^Cip1 ^acts as a negative regulator of the G_1_/S transition by inactivating the cyclin-dependent kinases that function to phosphorylate Rb and promote S phase entry [31].

Knockdown of FAS by siRNA or the small molecule inhibitor Orlistat induces apoptosis by activating caspase 8 (Knowles LM and Smith JW, unpublished observations). This is consistent with the current observation that siRNA targeting FAS up-regulates TNF-α and TRAIL death receptor mediated apoptosis pathways. In addition to *TRAIL*, cell death through FAS inhibition has also been linked to up-regulation of *BNIP3 *and *DAP Kinase 2 *[17]. These two proapoptotic genes were detected by microarray analysis 72 h after FAS inhibition, which explains why we did not observe similar changes as our study was limited to 48 h. A possible mechanism accounting for the ability of FAS inhibition to induce these apoptotic effects is the accumulation of ceramide, which has been shown to induce these death receptor pathways [32]. Ceramide accumulation is the result of malonyl-CoA build-up following inhibition of FAS [17]. Importantly, we have found that knockdown of FAS up-regulates *SGPL1*. Ceramide generation and promotion of cellular apoptosis have been observed in response to increased expression of SGPL1 [18], suggesting this enzyme may represent a leverage point by which knockdown of FAS influences ceramide production.

In addition to changes in proliferation and survival, knockdown of FAS also suppresses genes in the glycolysis, krebs-TCA cycle and oxidative phosphorylation metabolic pathways suggesting an overall reduction in cellular energy metabolism. Consistent with the down-regulation of oxidative phosphorylation gene expression, Chajès et al. [33] report impaired mitochondrial function and generation of reactive oxygen species in response to FAS inhibition. Targeted inactivation of ATP citrate lyase, a lipogenic enzyme which functions immediately up-stream FAS, has the opposite effect by stimulating mitochondrial function [34]. This is interesting as we have found that knockdown of FAS, but not of ATP citrate lyase, induces apoptosis (Knowles LM and Smith JW, unpublished observations); raising the question of whether the changes in cell survival and metabolism are linked together. Indeed, growing evidence suggests TNF-α may influence metabolism, as it can induce the down-regulation of genes involved in oxidative phosphorylation [35], inhibition of electron transport chain activity [36] and generation of reactive oxygen species [37]. Identifying whether induction of apoptosis and reduced energy metabolism are connected (via TNF-α or another molecular target) will be an important direction for future investigations, and may prove critical for understanding the anti-tumorigenic benefits of a knockdown in tumor FAS.

The overall goal of our study was to define the regulatory changes that take place following inhibition of tumor FAS. To this end, we have identified a number of target genes specifically modified by knockdown of FAS. Of particular interest among these are the dominant negative HLH transcription repressors ID2 and ID3, which function to bind and inactivate the basic HLH family of transcription factors [38], and therefore, are recognized for their ability to block cell differentiation and promote proliferation [39]. Like FAS, ID over-expression correlates with tumor development in an array of cancers [40,41], and knockdown of ID2/ID3 induces growth arrest and apoptosis [39,42]. Our finding that knockdown of FAS leads to the suppression of transcripts encoding *ID2 *and *ID3 *provides evidence of a link between FAS and ID expression. This link is further supported by observations that ID2/ID3 proteins can also suppress FAS gene expression by inactivating the basic HLH transcription factor SREBP1 c that controls FAS expression [21]. Further characterization of the molecular interplay between FAS and ID is needed to more fully understand the role of these in tumorigenesis.

## Conclusion

In conclusion, this study provides a comprehensive database of genomic changes that occur in response to knockdown of FAS and confirms that FAS is central not only for tumor cell metabolism but also for tumor cell signaling. Further characterization of these signals on the protein level will offer a more complete understanding of the cellular targets and pathways affected by knockdown of FAS and may lead to new therapeutic strategies for tumor prevention and treatment.

## Methods

### Cell line and culture conditions

The MDA-MB-435 mammary tumor cell line was obtained from Janet Price at the University of Texas Southwestern. MDA-MB-435 cells were maintained in Minimum Essential Medium Eagle (MEM) with Earl's Salts (Mediatech, Inc., Herndon, VA, USA) supplemented with 10% fetal bovine serum (Irvine Scientific, Santa Ana, CA, USA), 2 mM L-glutamine (Invitrogen Life Technologies, Inc., Carlsbad, CA, USA), MEM vitamins (Invitrogen Life Technologies, Inc.), nonessential amino acids (Mediatech, Inc.) and antibiotics (Omega Scientific, Inc., Tarzana, CA, USA). Cells were grown at 37°C under a humidified, 5% CO_2 _atmosphere.

### FAS gene silencing using siRNA

Four individual FAS siRNA sequences corresponding to 5'-GAGCGUAUCUGUGAGAAACUU-3' (nucleotides 6241–6259; FAS#1), 5'-GACGAGAGCACCUUUGAUGUU-3' (nucleotides 1741–1749; FAS#2), 5'-UGACAUCGUCCAUUCGUUUUU-3' (nucleotides 1758–1776; FAS#3), 5'-UGACAUCGUCCAUUCGUUUUU-3' (nucleotides 6236–6254; FAS#4) were designed and synthesized by Dharmacon (Lafayette, CO, USA). MDA-MB-435 cells were plated at 7.81 × 10^3 ^cells/cm^2 ^in 10-cm^2 ^plates and grown for 24 h prior to transfection with 25 nM FAS#1, FAS#2, FAS#3, FAS#4 or non-silencing control duplex #2 (D-001210-02) siRNA in Opti-MEM medium (Invitrogen) using Lipofectamine 2000 reagent (Invitrogen). After 5 h, transfection medium was replaced with normal culture medium and cells were grown for 12–48 h post-transfection.

### Real-time quantitative PCR

Total RNA was isolated from adherent cells using the Qiagen RNeasy kit (Qiagen, Inc, Valencia, CA, USA). Genomic DNA contamination was removed using RNase-Free DNase (Qiagen). RNA purity was assessed by A_260_/A_280 _absorption and RNA integrity was verified by agarose gel electrophoresis. cDNA was synthesized from 4 μg RNA using the Superscript III First-Strand Synthesis System (Invitrogen). Real-time quantitative PCR reactions were performed using Power SYBR^® ^Green PCR Master Mix (Applied Biosystems, Foster City, CA, USA) on a Stratagene Mx3000p QPCR System (La Jolla, CA, USA). cDNA was amplified using forward and reverse primers for FAS (5'-AACTCCATGTTTGGTGTTTG-3' and 5'-CACATGCGGTTTAATTGTG-3') and normalized to the housekeeping gene P0 (5'CAAGACTGGAGACAAAGTGG-3' and 5'AATCTGCAGACAGACACTGG-3'). The formation of a single PCR product was verified using automated melting curve analysis.

### Western blot analysis

Adherent cell populations were lysed in 2× SDS sample buffer, separated by SDS-PAGE and transferred to nitrocellulose as previously described [9]. Membranes were probed for FAS and β-tubulin protein expressions using anti-FAS (BD Transduction Laboratories, San Jose, CA, USA) and anti-β-tubulin (Upstate Biotechnology, Lake Placid, NY, USA) antibodies. Immunoreactivity was detected using anti-mouse IgG conjugated peroxidase and visualized by enhanced chemiluminescence.

### FAS activity

FAS activity was determined by measuring the incorporation of [^14^C]malonyl-CoA into cellular fatty acids as previously described [9]. Briefly, MDA-MB-435 cells were lysed in 20 mM Tris (pH 7.5), 1 mM dithiothreitol, and 1 mM EDTA by sonication. Insoluble material was removed by centrifugation (14,000 rpm) for 15 min at 4°C. The resulting lysates (70 ug) were incubated in reaction buffer containing 500 μM NADPH, 166.6 μM acetyl CoA, 100 mM KCl and 0.4 μCi [^14^C]malonyl-CoA (GE Healthcare, Piscataway, NJ, USA) for 25 min at 37°C. Reactions were chased with 25 nM cold malonyl-CoA for 15 min and terminated by the addition of chloroform:methanol (1:1). The chloroform extract was dried under N_2 _and extracted with water-saturated butanol. The butanol extract was evaporated under N_2_, and labeled fatty acids were detected using a Beckman model LS 5000CE Scinillation Counter (Beckman Coulter, Inc., Fullerton, CA, USA).

### BeadArray gene expression

MDA-MB-435 cells were transfected with siRNA on two separate occasions to generate independent measurements for each treatment, and monitored for whole genome expression at 12 h intervals over a 48 h time period. Total RNA was isolated and examined for purity/integrity as described above. Labeled cRNA was prepared from 500 ng RNA using the Illumina^® ^RNA Amplification Kit from Ambion (Austin, TX, USA). The labeled cRNA (1500 ng) was hybridized overnight at 55°C to the Sentrix^® ^HumanRef-6 Expression BeadChip (>46,000 gene transcripts; Illumina, San Diego, CA, USA) according to the manufacturer's instructions. BeadChips were subsequently washed and developed with fluorolink streptavidin-Cy3 (GE Healthcare). BeadChips were scanned with an Illumina BeadArray Reader and hybridization efficiency was monitored using BeadStudio software (Illumina) to measure internal controls built into the Illumina system.

### Expression data analysis

Expression data was filtered to identify genes expressed on the BeadChip at >0.99 confidence (16,585 genes), and normalized per chip using non-linear Normalize.quantiles or VSN normalization to remove array to array variability (Bioconductor Project [43]). The efficiency of both normalization methods were validated by measuring gene to gene correlations across BeadChips according to the methods of Ploner et al. [44] using R 2.0.1 software (R Development Core Team, Vienna, Austria).

Differentially expressed genes that changed over time in response to each of the four FAS siRNA duplex were identified using two independent methods. In the first method, per chip normalized Normalize.quantiles data was imported into the GeneSpring GX 7.3.1 software package (Agilent Technologies, Palo Alto, CA, USA), and per gene normalized to the appropriate biological control for each time point. Genes that significantly changed over time in response to each FAS siRNA duplex were compared against control using a two-way ANOVA with a Benjamini & Hochberg FDR of 0.05. In the second method, VSN normalized data was imported into the Bioconductor Timecourse package [43]. Genes regulated over time by each FAS siRNA duplex in both biological replicates were determined using mb. long() statistics, with an mb>0 used as criterion for the identification of significantly regulated genes. Genes identified as significant using these two complementary approaches were combined, and genes with a *p*-value ≤ 0.05 and with 1.2 fold changes in both the biological replicates of each duplex at a given time point, were maintained for expression analysis. Genes identified as significantly modified by each of the 4 FAS siRNA duplexes were then overlapped using venn diagrams to identify a set of genes modified by 3 or more of the siRNA treatments. Expression profiles of the core gene list were averaged, and genes modified by 1.5 fold were visually examined and ordered by hierarchical clustering using the Pearson Correlation similarity measurement (GeneSpring).

### Gene set enrichment analysis

For pathway analysis, data from the 16,585 genes expressed in MDA-MB-435 cells was exported from GeneSpring, filtered for duplicate symbols and analyzed using GSEA software (Broad Institute) according to published methods [26]. Briefly, data was overlapped on 522 gene sets (s2.symbols.gmt) downloaded with the GSEA package and measured for the enrichment of genes at the top or bottom of the gene list to determine their correlation with the gene set's phenotype. The GSEA parameters used included: metric for ranking genes, signal2noise; enrichment statistic, weighted; permutation type, phenotype; permutation number, 1000; and gene set size restrictions, 4 minimum, 500 maximum. Gene sets significantly modified by FAS siRNA treatment were identified using a nominal *p*-value < 0.05 and a multiple hypothesis testing FDR < 0.25. NES represents the enrichment of genes in the designated GSEA gene set, ranked according to the overrepresentation of genes at the top or bottom of the list, normalized to gene set size.

## Abbreviations

FAS, fatty acid synthase; GSEA, Gene Set Enrichment Analysis; HLH, helix-loop-helix; siRNA, small interfering RNA; SREBP, sterol regulatory element-binding protein; TRAIL, TNF-related apoptosis-inducing ligand.

## Authors' contributions

JS and LK conceived and designed the study. LK carried out the siRNA transfections, validated target knockdown, and prepared RNA for BeadArray expression profiling, performed data analysis and drafted the manuscript. JS supervised the coordination of the study and participated in manuscript preparation. All authors read and approved the final manuscript.

## Supplementary Material

Additional file 1 **Genes significantly modified by FAS siRNA duplex #1 (≥1.2 or ≤0.83 fold)**. The fold change values represent the average from 2 replicates of FAS siRNA duplex #1 compared to non-silencing control siRNA. Significance was determined using a *p*-value of < 0.05, with a 1.2 fold change cutoff for both biological replicates at a given time point. Click here for file

Additional file 2 **Genes significantly modified by FAS siRNA duplex #2 (≥1.2 or ≤0.83 fold)**. The fold change values represent the average from 2 replicates of FAS siRNA duplex #2 compared to non-silencing control siRNA. Significance was determined using a *p*-value of < 0.05, with a 1.2 fold change cutoff for both biological replicates at a given time point. Click here for file

Additional file 3 **Genes significantly modified by FAS siRNA duplex #3 (≥1.2 or ≤0.83 fold)**. The fold change values represent the average from 2 replicates of FAS siRNA duplex #3 compared to non-silencing control siRNA. Significance was determined using a *p*-value of < 0.05, with a 1.2 fold change cutoff for both biological replicates at a given time point. Click here for file

Additional file 4 **Genes significantly modified by FAS siRNA duplex #4 (≥1.2 or ≤0.83 fold)**. The fold change values represent the average from 2 replicates of FAS siRNA duplex #4 compared to non-silencing control siRNA. Significance was determined using a *p*-value of < 0.05, with a 1.2 fold change cutoff for both biological replicates at a given time point. Click here for file

Additional file 5 **FAS knockdown signature of 279 genes modified by 1.5 fold**. The table displays genes modified by 1.5 fold compared to non-silencing control siRNA. The fold change values represent the average from at least 3 of the FAS siRNA duplexes. Click here for file

Additional file 6 **GSEA pathways down-regulated in response to knockdown of FAS**. The table displays all pathways found to be down-regulated in response to FAS siRNA treatment compared to non-silencing control siRNA. Significance was determined using a nominal *p*-value < 0.05 or FDR < 0.250. Click here for file

Additional file 7 **GSEA pathways up-regulated in response to knockdown of FAS**. The table displays all pathways found to be up-regulated in response to FAS siRNA treatment compared to non-silencing control siRNA. Significance was determined using a nominal *p*-value < 0.05 or FDR < 0.250. Click here for file
